# Vertically Aligned Carbon Nanotube Mechano‐Electrochemical Generator for Ultralow‐Frequency Ocean Wave Monitoring

**DOI:** 10.1002/advs.202503578

**Published:** 2025-05-05

**Authors:** Hyeon Jun Sim, Dong Yeop Lee, Hocheol Gwac, Seungjin Lee, Joonhyeon Jeon, Seon Jeong Kim, Young‐Kwan Kim, Chang‐Seok Kim, Young‐Jin Kim, Sooncheol Kwon, Changsoon Choi

**Affiliations:** ^1^ Department of Biomedical Engineering Konkuk University Chungju 27478 South Korea; ^2^ Department of Electronic Engineering and Biomedical Engineering Hanyang University Seoul 04763 South Korea; ^3^ Department of Advanced Battery Convergence Engineering Dongguk University‐Seoul Seoul 04620 South Korea; ^4^ Department of Electronic and Electrical Engineering Dongguk University‐Seoul Seoul 04620 South Korea; ^5^ Department of Chemistry Dongguk University‐Seoul Seoul 04620 South Korea; ^6^ Department of Cogno‐Mechatronics Engineering Pusan National University Geumjeong‐gu Busan 46241 Republic of Korea; ^7^ Medical Device Development Center Osong Medical Innovation Foundation Cheongju Chungbuk 28160 South Korea; ^8^ Department of Energy and Materials Engineering Dongguk University‐Seoul Seoul 04620 South Korea

**Keywords:** Carbon nanotube forest, Low frequency, Mechano‐electrochemical energy harvester, Self‐powered sensor, Wave monitoring

## Abstract

Ultralow‐frequency waves contain crucial information related to natural disasters; however, conventional technologies are limited in their ability to measure them accurately. To address this challenge, a novel vertically aligned CNT mechano‐eletrochemical generator is proposed that generates electrical energy via fluctuation in the electrode‐electrolyte interface. This generator utilizes ion movement based on electrochemical interactions, enabling it to generate electrical energy even in ultralow‐frequency environments like ocean waves. In addition, the direct contact between the electrolyte and CNTs prevents signal degradation or distortion caused by packaging, allowing the precise detection of complex waveforms with overlapping frequencies. Given these characteristics, the generator exhibits broad applicability in complex environments, such as ocean monitoring. Furthermore, it demonstrates significant potential for future applications, such as self‐powered oceanographic sensors and sustainable energy harvesting.

## Graphical abstract

A mechano‐electrochemical generator is proposed that harvests energy from ultralow‐frequency waves below 0.01 Hz for self‐powered disaster monitoring. The generator minimizes signal distortion, enabling precise measurement of subtle waves with overlapping frequencies. It successfully generated electrical energy from actual ocean waves and analyzed the frequency data using FFT.

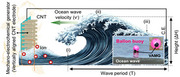

## 1 Introduction

Blue energy resources, such as ocean waves and currents, represent a virtually inexhaustible reservoir of energy while simultaneously containing valuable environmental data.^[^ ^1^, ^2^, ^3^, ^4^ ^]^ Hence, generating electrical energy from blue energy, such as ocean waves, has garnered significant attention in several studies. In addition, research is being conducted to predict and mitigate natural disasters such as earthquakes and tsunamis by analyzing the amplitude and frequency of waveforms.^[^ ^5^, ^6^ ^]^ Accordingly, several sensor mechanisms have been developed to monitor ocean waves, each providing different advantages and disadvantages in monitoring performance. Global positioning systems (GPS) track geological instability signs on land, such as terrain shifts, rock movement, and ground uplift. However, as a GPS systems communicate via microwave frequencies, water efficiently absorbs these signals, making it difficult to monitor ocean surface dynamics.^[^ ^7^ ^]^ Alternatively, resistive and capacitive sensors are prevalent in wave monitoring.^[^ ^8^, ^9^ ^]^ However, these sensors require external power supplies, such as batteries, which presents limitations for ocean wave monitoring as deployment across expansive areas necessitates frequent maintenance, posing logistical challenges.

In recent years, the piezoelectric and triboelectric energy harvesting technologies have significantly advanced the development of self‐powered sensors, offering the capability to convert mechanical energy into electrical signals and thereby reducing reliance on external power sources.^[^ ^10^, ^11^ ^]^ Despite these advances, critical limitations remain when such technologies are applied to ocean wave monitoring. Ocean waves typically consist of complex superimpositions of multiple frequency components ranging broadly from 0.01 Hz to several 0.5 Hz.^[^ ^5^, ^6^ ^]^ However, existing energy harvesting mechanisms based on piezoelectric and triboelectric effects are inherently optimized for high‐frequency mechanical inputs.^[^ ^12^, ^13^ ^]^ As a result, their electrical outputs are often confined to narrow pulse widths in the microsecond range, making them unsuitable for detecting the slow, low‐frequency wave motions that dominate marine environments. This frequency incompatibility poses a fundamental barrier to the detection and prediction of catastrophic natural events such as tsunamis and underwater earthquakes, which often exhibit characteristic frequencies below 1 Hz. Although certain design modifications have been proposed to lower the operational frequency thresholds, these approaches frequently rely on complex device architectures or specialized structural constraints, limiting their scalability and real‐world deployment potential.

Another significant obstacle to the deployment of conventional self‐powered sensors in marine applications lies in their vulnerability to water exposure. The charge accumulation mechanisms inherent to these devices are highly susceptible to disruption in humid or submerged environments, where water molecules facilitate rapid charge dissipation at the electrode surface.^[^ ^14^ ^]^ This environmental instability severely compromises both sensitivity and operational reliability. While encapsulation strategies have been explored to mitigate water ingress, such protective packaging typically results in increased sensor weight and volume, as well as diminished sensitivity, thereby only partially resolving the issue.

To overcome these limitations, we introduce a mechano‐electrochemical energy harvesting approach tailored specifically for low‐frequency, water‐immersed applications. This method leverages the modulation of the electrical double‐layer capacitance (EDLC) formed at the interface between an electrode and an electrolyte, enabling direct conversion of mechanical wave motion into electrical signals. Our previous studies have shown that mechanical deformation of carbon nanotube (CNT)‐based working electrodes immersed in an electrolyte can induce reproducible, low‐frequency voltage signals in the range of several hundred millivolts.^[^ ^15^, ^16^, ^17^, ^18^, ^19^, ^20^ ^]^ Distinct from prior studies that focused on the electrode side, the present work shifts the focus to the electrolyte, namely, the ocean wave itself as the dynamic component inducing capacitance changes. In this configuration, the continuous variation in ion distribution at the electrode surface caused by wave‐driven changes in contact area generates electrical signals with operational frequencies as low as 0.02 Hz, enabling real‐time monitoring of ocean wave dynamics under ultra‐low frequency conditions. Moreover, this mechanism is inherently water‐compatible and does not require any packaging, allowing for direct contact between the sensor and the ocean environment without sacrificing sensitivity.

**Figure 1:**
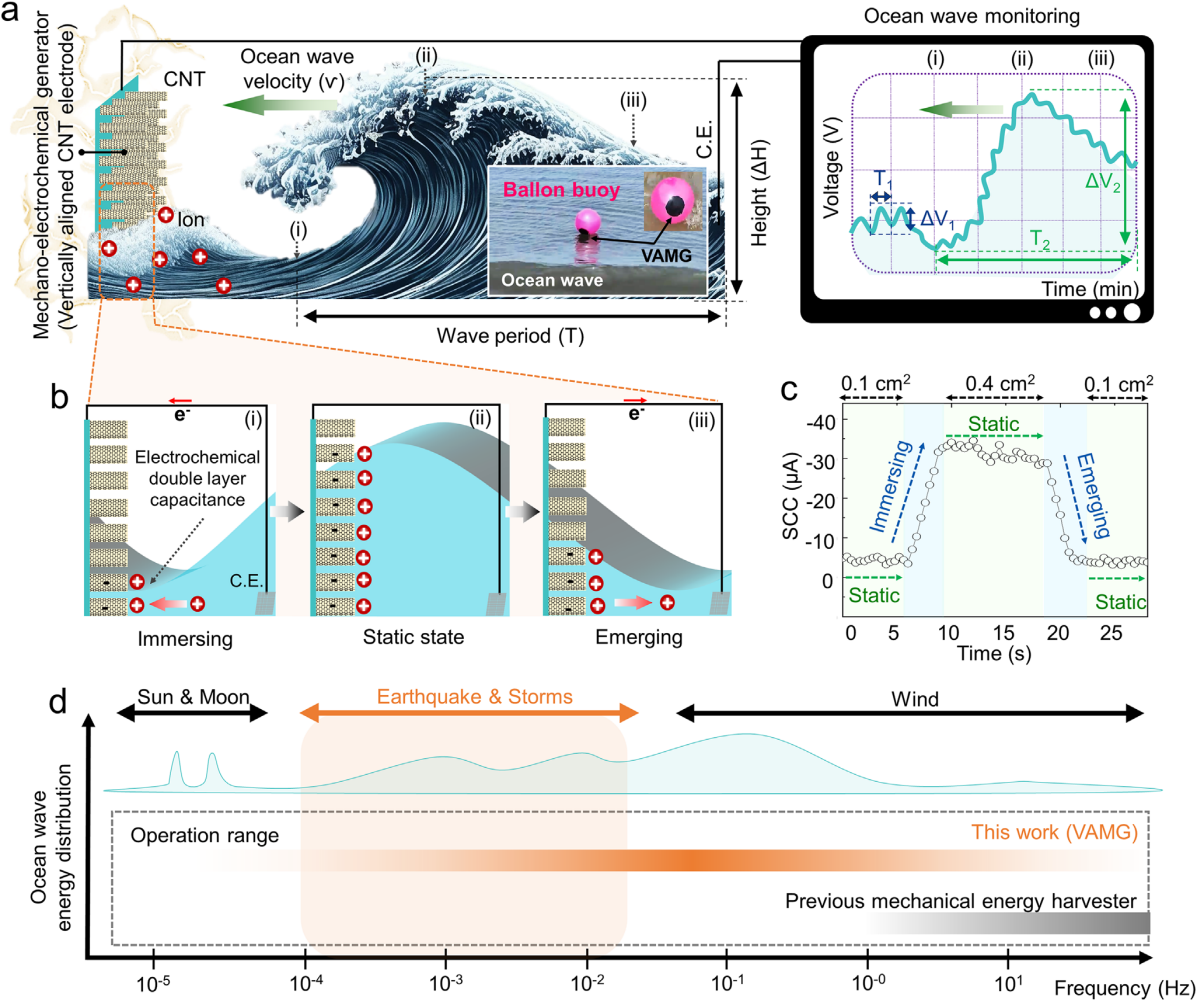
a) Schematic illustration of the electrical generation principle of the mechano‐electrochemical generator. When waves triggered alterations in the electrode‐electrolyte interface, the generator's potential difference was induced accordingly. This generates electrical energy from waves and enables self‐powered wave height and frequency sensing as electrical signals without requiring an external power source. (Inset) Optical image of the ocean monitoring system with a VAMG attached to the surface of a balloon buoy, which moves according to the wave motion. b) Schematic diagram of the generation principle. As the electrode‐electrolyte interface increases due to waves, the electrochemical double‐layer capacitance formed between the electrode and electrolyte also increases, leading to a potential difference in the electrode. c) SCC values generated by VAMG when subjected to a square wave stimulus with a 0.3‐cm height. d) Graph showing the distribution of ocean wave energy across frequencies. (Inset) A comparison graph displays the frequency range of the proposed VAMG and conventional mechanical energy harvesters. Because of its superior sensitivity at extremely low frequencies, VAMG outperforms conventional technologies by covering a wider frequency range in both energy harvesting and wave monitoring applications.

As a novel electrode platform, we propose a 2D, vertically aligned carbon nanotube (VACNT) array, which offers exceptional electrochemical surface area, intrinsic electrical conductivity, chemical stability, and structural scalability. The resulting vertically aligned mechano‐electrochemical generator (VAMG) translates fluctuations at the electrode‐electrolyte interface into electrical outputs (**Figure** **1**a). The VACNT forest provides an intrinsic bias voltage without the need for external power sources, allowing stable and repeatable signal generation. Its accordion‐like structure offers both high areal capacitance and mechanical flexibility, optimizing energy output while ensuring environmental adaptability. The VAMG's operation hinges on modulation of interfacial capacitance and electrochemical potential in response to wave‐induced motion (Figure 1b). The inherently slow ion diffusion processes contribute to long‐lasting current generation up to 10 s even in quasi‐static conditions, contrasting sharply with the millisecond‐scale signal widths typical of conventional piezoelectric or triboelectric systems (Figure 1c). The proposed system achieves an open‐circuit voltage of 350 mV, a short‐circuit current of 190 µA, and a peak power density of 6 W m^−^^2^, outperforming existing mechano‐electrochemical harvesters. The VAMG effectively detects both artificial and natural waveforms, including sinusoidal and complex interfering patterns, enabling accurate sensing of ultralow‐frequency signals associated with seismic and tidal events (Figure 1d).

## 2 Results and Discussion

### 2.1 Fabrication Process and Characteristics of the Proposed VAMG System

**Figure 2:**
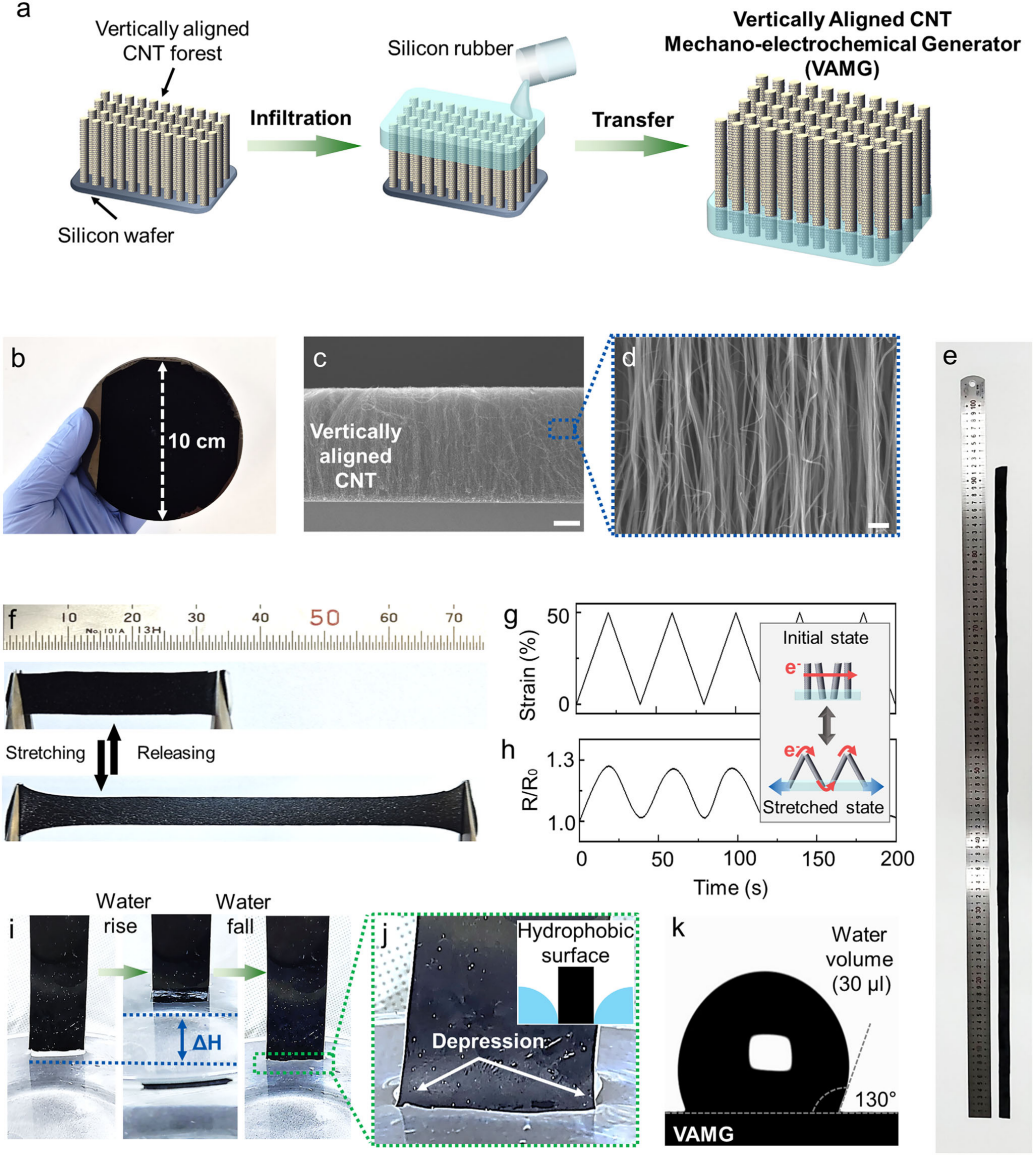
Morphology and characteristics of proposed VAMG. a) Schematic illustration of the fabrication process of proposed VAMG. b) Optical image, c) SEM image, and d) magnified SEM image of a vertically aligned CNT forest. e) Optical image of a 90‐cm scaled‐up VAMG connected electrically. f) Optical image of VAMG at 0% strain (initial state) and 100% strain (stretched state). g) Repeated application of 50% strain to proposed VAMG and h) corresponding variation in resistance relative to its initial resistance. i) Optical image and j) magnified optical image of the VAMG when the water level fluctuates. No residual water droplet was observed on the VAMG surface after reversible immersion. k) Contact angle between the 30‐µL water droplet and VAMG surface.

The proposed VAMG was fabricated via a simple sequential process (**Figure** **2**a). First, the CNT forest is fabricated using a chemical vapor deposition method on a silicon wafer, which forms a cylindrical structure with a diameter of 10–15 cm (Figure 2b; Figure S1, Supporting Information).^[^ ^21^ ^]^ The CNTs, each with 7–8 walls, have an average length of 400 µm. Owing to the high alignment and bundling of the CNT, the forest structure enables spinnable sheets that are both electrically conductive and mechanically deformable (Figure S2, Supporting Information). In addition, the vertically aligned CNT forest structure maximizes the number of CNTs per unit area (≈10^9^ CNTs mm^−^^2^) and enables a highly porous structure.

Subsequently, to fabricate the VAMG, a solution of silicone rubber and curing agent mixed in a 1:1 ratio is evenly applied to the CNT forest surface. Owing to the high viscosity of the silicone rubber, some of the solutions infiltrate between the CNTs and solidifies, forming a VAMG with an average thickness of ≈1.2 mm (Figure S3, Supporting Information). When the VAMG is peeled off from the substrate, the CNT structure is transferred by the infiltrated silicone rubber, maintaining the vertical alignment of the nanostructure (Figure 2c,d). The VAMG has a unique asymmetric structure, with one side exposed to the CNTs, and another encapsulated in silicon rubber (Figures S4 and S5, Supporting Information). In addition, it can be cut or designed into desired shapes and electrically connected via the fabrication process and structure to enable scaling up to lengths exceeding 90 cm (Figure 2e).

The vertically aligned CNT forest structure and silicone rubber make the proposed VAMG soft and stretchable. According to the strain‐stress curve, the proposed VAMG exhibits remarkable flexibility. With Young's modulus of 5.5 kPa, it can be stretched to over 100% and still maintain its elastic properties (Figure 2f; Figure S6a, Supporting Information). Its flexibility and stretchability allow it to be attached to curved surfaces like the spherical surface of a buoy balloon (Inset of Figure 1a). Notably, the adhesion coefficient of silicone rubber allows it to adhere well to surfaces without requiring adhesive. In addition, by cutting the VAMG into desired shapes, various electrode configurations can be easily mass‐produced. Ribbon‐shaped electrodes with a 1‐mm width can be utilized as stretchable electrodes capable of bending, twisting, and stretching (Figure S6b, Supporting Information).

Furthermore, due to the vertically aligned CNT forest structure, the proposed VAMG demonstrates stable electrical performance in mechanical deformation, exhibiting characteristics of stretchable electrodes. With a 1‐cm width and 1‐cm length, the VAMG's initial resistance is 98 kΩ. When the strain gradually increases from 0% to 50%, the resistance value increases 1.3 times compared to the initial resistance; however, the electrical conductivity remains unchanged (Figure 2g,h). The CNTs of the proposed VAMG are electrically connected, and when stretched, they form an accordion‐like structure, maintaining the electrical pathways.^[^ ^22^ ^]^ During this process, there is a tendency for resistance to increase due to partial disconnections in the electrical connections; however, when the strain returns to 0%, the broken electrical connections are restored, and resistance decreases. Mechanical energy generators require mechanical and electrical stability to generate electrical energy while ensuring consistent performance under extreme deformation. The proposed generator maintains high mechanical resilience and electrical characteristics even under high stretching of over 50%, ensuring the stability of the device even under ocean conditions and wave‐induced deformations.

In addition, owing to the VAMG's material and structural characteristics, it exhibits excellent hydrophobic properties. CNT and silicone rubber are inherently hydrophobic,^[^ ^23^ ^]^ and the vertically aligned nano‐structure of the CNTs further enhances this hydrophobic effect. Consequently, even after repeated immersion in electrolytes, water droplets do not form on the non‐immersed electrode surface (Figure 2i). Furthermore, due to the hydrophobic characteristics at the interface with the electrolyte, the VAMG surface causes depression to slightly curl inward (Figure 2j). In previous studies where electricity was generated via alterations in the electrode‐electrolyte interface, hydrophilic electrodes posed several challenges.^[^ ^24^ ^]^ Because of their hydrophilic properties, residual moisture remained on the electrode surface even after they emerged from the electrolyte. This resulted in minimal variation in the electrode‐electrolyte interface despite water level alterations. Previous research required additional drying to eliminate residual moisture from the electrode surface after each immersion to maximize this change. This significantly limited continuous energy harvesting and self‐powered wave‐sensing applications. By harnessing the highly hydrophobic properties of the proposed VAMG (contact angle of 130 degrees, Figure 2k), we designed the device such that only the immersed portion of the electrode forms a contact area with the electrolyte. Consequently, no residual moisture remained on the surface after emergence, eliminating the need for an air‐drying process.

### 2.2 Electricity Generation Mechanism Based on a Mechano‐Electrochemical Principle

**Figure 3:**
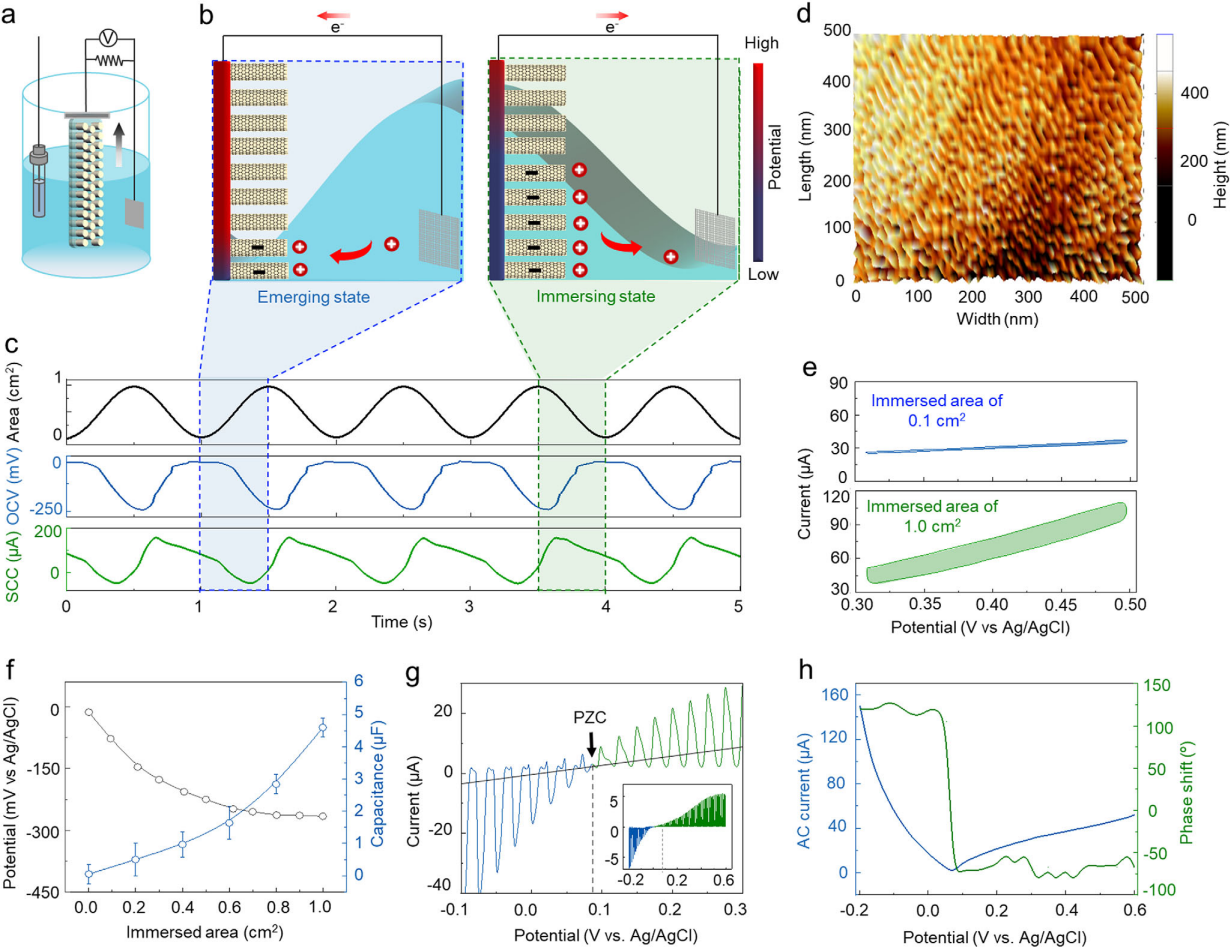
Electrical generation principle of VAMG. a) VAMG, counter, and reference electrodes in an electrolyte solution, illustrating the measurement setup in response to water level fluctuation. b) Schematic illustration representing the principle of potential difference as the immersed surface area of the proposed VAMG. Illustration presenting the relationship between the immersed area, electrochemical capacitance, and potential difference. As the immersed surface area of VAMG increases, the electrochemical double‐layer capacitance increases, leading to a corresponding potential difference in the electrode. c) Proposed VAMG generated OCV and SCC values as they were repeatedly immersed in 0.6 m NaCl electrolyte with 1‐Hz frequency. d) AFM image of the VAMG surface showing the alignment of CNTs in a vertically oriented structure. e) Cyclic voltammograms of the VAMG electrode in 0.6 m NaCl with an immersed area of 0.1 and 1 cm^2^ (scan rate: 100 mV s^−1^). f) Capacitance and OCV values of VAMG based on the immersed area. g) Cyclic voltammograms of the VAMG electrode in 0.6 M NaCl under 1‐Hz sinusoidal deformation with a height of 1 cm (blue and green lines) and without deformation (black) (scan rate: 10 mV s^−1^). h) Magnitude and phase of AC current relative to the mechanical amplitude. The potential of minimum current amplitude and 180° phase shift corresponds to the potential of zero charges (PZC, 100 mV vs. Ag/AgCl) divided by the initial resistance) of the VAMG during repeated stretching over time.

The proposed VAMG, which generates electrical energy in response to alterations in the water level, can be adopted as a wave‐energy generator and self‐powered wave sensor. To verify this assertion, we employed a three‐electrode system, where the VAMG, a Pt mesh, and an Ag/AgCl electrode functioned as the working, counter, and reference electrodes for electrochemical measurements, respectively (**Figure** **3**a). The observed VAMG, which was 1‐cm in width and 5‐cm in length, was immersed in a 0.6M NaCl solution (mimicking ocean applications) with a sinusoidal adjustment of the immersed area to a 1‐cm height at a 1‐Hz frequency.

As the immersed area of the VAMG periodically fluctuated in the electrolyte, it proportionally generated electrical energy. When the VAMG was nearly fully immersed (at ≈1 cm^2^), the OCV dropped to −250 mV (Figure 3b). Conversely, as the immersed area decreased, the potential was observed to recover repeatedly. Simultaneously, the SCC varied by altering the electrode‐electrolyte interface, thus generating a maximum of 190 µA. To verify that the generated electricity was from the VAMG itself, a switching polarity test was conducted (Figure S7, Supporting Information). By reversing the connections on the oscilloscope, we confirmed that the waveform and direction of the generated voltage were reversed.

The coupling between electrochemical capacitance and electrical potential can explain the mechanism of electricity generation due to changes in surface area. When a carbon nanotube electrode is immersed in an electrolyte, a potential is generated at the interface between the electrode and the electrolyte, which induces charge accumulation. As the contact area between the CNTs and the electrolyte increases, the area over which electrochemical electric double layers are formed also increases, thereby increasing the ion‐accessible surface area (Figure 3c). Consequently, the electrochemical capacitance of increases with the expansion of the electrode–electrolyte interface in the VAMG. For example, when the immersed area is 0.1 cm^2^, the capacitance is 0.5 µF (1.7 F kg^−1^). Accordingly, when the area increases to 1.0 cm^2^, the capacitance increases to 4.8 µF (16 F kg^−1^) (Figure 3d,e; Figure S8, Supporting Information). When the CNT electrode is immersed in an electrolyte, a potential is formed between them, which induces an electric charge according to Equation (1):^[^ ^15^ ^]^

(1) Q=CV=C(OCV−PZC)

where Q corresponds to the charge on the surface, C denotes the capacitance of the fiber, V is the intrinsic voltage, OCV is the open‐circuit voltage and PZC denotes the potential of zero charge. According to Equation (1), a change in the electrode capacitance leads to a variation in the electrical output (Figure S9, Supporting Information).

When the electrode is immersed, we must determine the potential of zero charge (PZC) to evaluate its equilibrium charge state. To determine PZC, we employed piezo electrochemical spectroscopy (PECS), a technique that relies on the charge‐state‐dependent response of the electrode to mechanical stimuli. PECS involves characterizing the electrode with cyclic voltammetry (CV) while subjecting it to sinusoidal immersion. By comparing CV scans with and without immersion, we can determine the magnitude and phase dependence of the alternating current (AC) induced by mechanical stimulation. PZC is the potential obtained when the AC is minimized, and the current phase shifts 180° (Figure 3f,g). For the VAMG in a 0.6M NaCl solution, the PZC was determined to be 0.09V. The OCV formed by the VAMG upon immersion in the electrolyte was negative relative to the Ag/AgCl reference, indicating that the potential was lower than the PZC, causing a primary interaction with cations on the CNT surface.

In previous studies that generated electricity via alterations in capacitance, an external bias voltage was required to induce electrochemical capacitance.^[^ ^25^ ^]^ However, this increased the overall complexity and volume of the device and required continuous maintenance, such as periodic recharging or power supply replacement. In contrast, the proposed VAMG forms an intrinsic bias voltage (OCV‐PZC) due to the CNT.^[^ ^15^ ^]^ This allows for self‐sustained energy generation without requiring an external power supply, making the device highly suitable for remote or inaccessible energy harvesting and environmental monitoring applications. In addition, we confirmed that the electrical performance varies depending on the polymer coated on the CNT forest. Supporting these findings, when hydrophobic polymers such as PCL, PVDF, SEBS, and silicone rubber were utilized to form CNT forest/polymer composites, it was observed that silicone rubber exhibited the highest contact angle and peak voltage (Figures S10 and S11, Supporting Information), and this was subsequently employed in future experiments.

### 2.3 VAMG Performance for Measuring Ultralow‐Frequency and Composite Wave

**Figure 4:**
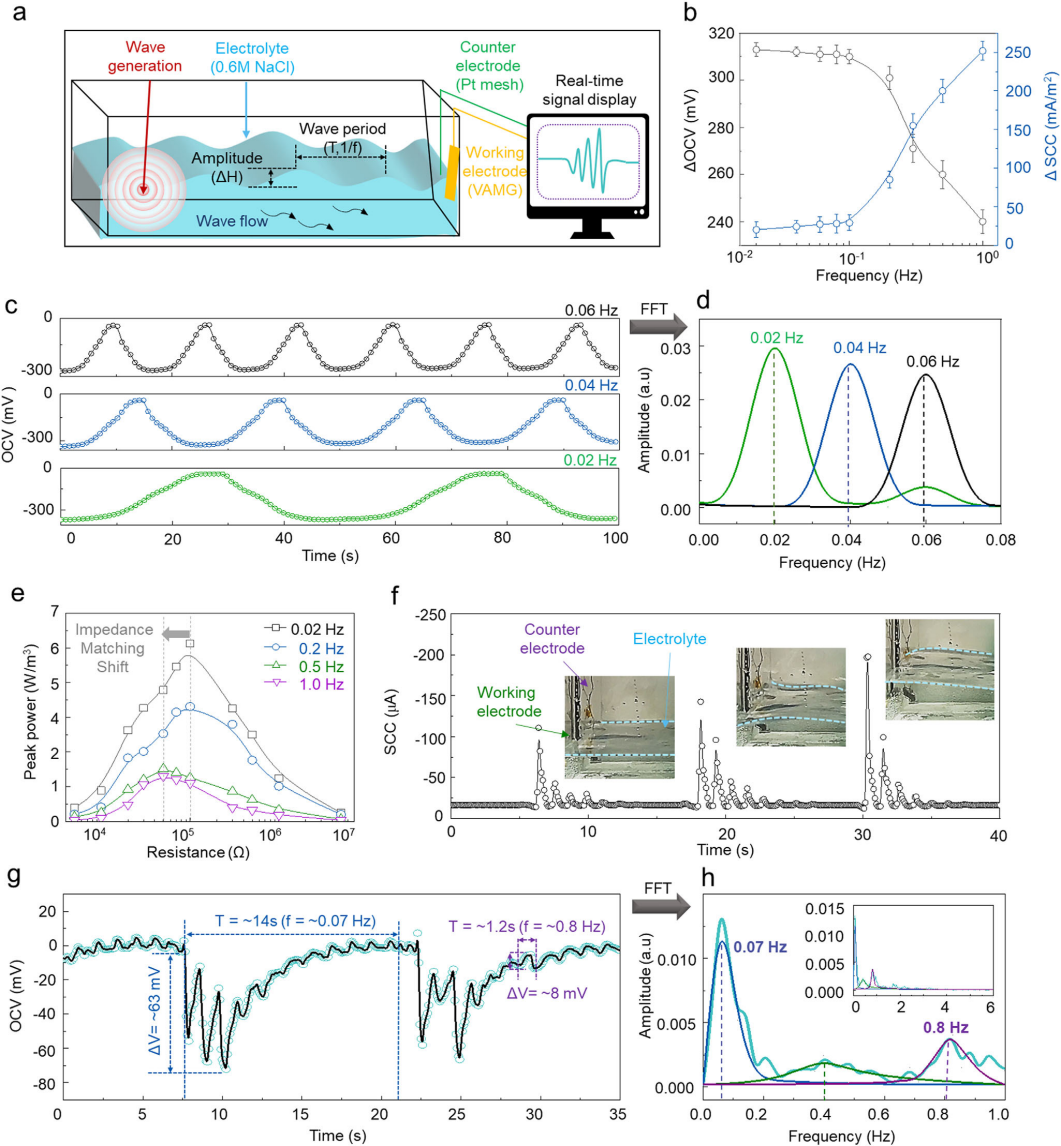
Self‐powered wave sensing performance in artifical wave. a) Schematic diagram of the experimental setup employed to measure the electrical output characteristics of VAMG using artificial waves. OCV and SCC generated by the VAMG with 1‐cm width and 7.5‐cm height dimensions in a 0.6 m NaCl electrolyte are measured. b) OCV and SCC values are generated by the VAMG in response to frequency variations from 0.01 to 1 Hz when a sinusoidal wave with 1.2‐cm height emerges. c) Voltage graph generated by VAMG when subjected to low‐frequency (0.02, 0.04, and 0.06 Hz) sinusoidal mono‐wavelength waves, and d) the corresponding frequency domain analysis using FFT. e) Peak power generated by VAMG as a varying external resistance. f) SCC results measured by the VAMG in response to aperiodic, irregular waves, demonstrating its capability to measure temporal and spatial wave attenuation. g) OCV results are generated by VAMG when an interfering wave with various mixed frequencies, similar to real ocean waves, is applied. h) Corresponding frequency domain analysis using FFT to identify the components.

An artificial wave system was developed, and its ability to produce electrical energy in this environment was analyzed (**Figure** **4**a). Using a two‐electrode system, the working electrode was a 1 × 5 cm VAMG, and the counter electrode was a platinum mesh. When artificial waves were introduced in a 0.6 M NaCl electrolyte, the differences in voltage and current were observed using an oscilloscope and a source meter. When the electrode‐electrolyte interface fluctuated repeatedly at an ultralow frequency below 0.01 Hz, potential differences were observed according to the immersed area (Figure 4b,c; Figures S12 and S13, Supporting Information). For a sinusoidal artificial wave at a single frequency of 0.02, 0.04, and 0.06 Hz with a 1‐cm^2^ area change, an actual electrical output corresponding to the water level fluctuation was observed, indicating that electrical signals can identify the wave over time. Furthermore, a fast Fourier transformation (FFT) can analyze the wave's frequency domain (Figure 4d).

As the frequency increases from 0.01 to 1 Hz, the generated ΔOCV decreases from 310 to 240 mV, while the current increases from 20 to 250 mA m^−^^2^. The electrical impedance of the VAMG determines the generated output voltage and current. Although the VAMG's equivalent circuit is complex, a simple R‐C model can elucidate the primary characteristics. In this estimation, the impedance of the generator can be expressed according to Equation (2):^[^ ^15^ ^]^

(2) $$Z_{\mathrm{VAMG}}=R_{\mathrm{internal}}+1/\mathrm{jwC}$$

where j and ω denote the imaginary unit and angular frequency, respectively. At low deformation frequencies, due to the impedance of the double‐layer capacitance (Z_c_ =  1/jwC), the overall impedance decreases, resulting in a high voltage and low current. The capacitive impedance is minimized at higher frequencies, and low voltage is observed with high current.

A square wave step‐function‐like water level fluctuation was applied to verify the benefit of continuous operation from the slow ion diffusion dynamics (Figure S14, Supporting Information). When square wave‐type water level fluctuations were induced, we verified that in addition to the dynamic state, the current was also generated continuously for more than 10 s in proportion to the covered area in the static state. The VAMG based on electrochemical principle is optimized for continuous operation at low frequencies due to the slow dynamic movement of ions and the high charge density per unit area.^[^ ^26^, ^27^ ^]^ Compared to the millisecond peak width of triboelectric and piezoelectric generators, this represents a significant difference, enabling the measurement of responses to motion even in static conditions and making it applicable to various fields beyond energy harvesting.

To analyze the power of the generated electrical energy, external resistance was connected to the VAMG, and the voltage across the external resistance was measured to calculate the peak power (Figure 4d). At a 0.01‐Hz frequency, the highest peak power of 6 W m^−^^2^ was calculated at ≈100 kΩ. As the frequency increases, the volumetric and areal power decrease from 6 to 1.2 W m^−^^3^ and from 0.1 to 0.03 W m^−^^2^, respectively (Figure 4e). In addition, it was observed that the impedance matching value shifts to the right from 100 to 50 kΩ as frequency increases. This is because, as the frequency increases, the impedance due to the double‐layer capacitance decreases, reducing the overall impedance and decreasing the impedance‐matching resistance value.

The VAMG effectively measures both sinusoidal waves and aperiodic, irregular waves (Figure 4f; Movie S1, Supporting Information). When a transient mechanical impulse was applied to the opposite water surface at 10‐s intervals, waves are generated, which attenuate over time and distance. To generate interfering waves with multiple sine waves, a 30 × 80 cm tank was filled with an electrolyte to a 20‐cm height (Figure S17, Supporting Information). Artificial waves were generated using a fan 80 cm away to create wind and waves. These waves collided with the walls, overlapping multiple sine waves to form interfering waves. Waves with height and period of 1 cm and 0.1 Hz, respectively, were repeatedly applied. The proposed VAMG successfully captured the waveform of these waves as they diminished over time. Furthermore, as the intensity of the applied mechanical impulse increased, the wave height correspondingly increased, resulting in a proportional increase in the generated current from 50 µA to 190 µA.

Furthermore, the proposed VAMG accurately measures waves with two interfering frequencies (Figure 4g). Instead of single sinusoidal waves, actual ocean waves comprise interfering waveforms, and the precise measurement of these waveforms is crucial for an ocean monitoring sensor. To simulate this, interfering waves were generated artificially using wind and mechanical stimuli, and their subtle interfering wave was measured using the proposed VAMG. The electrical signal generated by the VAMG confirmed that a large, slow wave corresponding to an OCV of 63 mV and a relatively small, fast wave corresponding to an OCV of 8 mV interfered with each other. In addition, when the measured electrical signal was analyzed in the frequency domain using FFT, interference between waveforms with frequencies of 0.06 Hz and 0.8 Hz was observed (Figure 4i).

The amount of generated current was proportional to the surface area in contact with the electrolyte. As the width of the VAMG increased from 1 cm to 4 cm, the maximum current generated increased from 40 to 130 µA (Figure S15, Supporting Information). Furthermore, by connecting the system in series, the OCV value can be amplified to over 1.0 V (Figure S16, Supporting Information). To harness the generated electrical energy, it was stored in a commercially available 100‐µF capacitor (Figure S17, Supporting Information). Even after a sudden discharge, as in the case of powering an electronic device, the capacitor could be recharged with the generated electrical energy. This demonstrated the capability for actual energy measurement and the potential to utilize the generated energy as a power source for other electronic devices.

Furthermore, for the proposed VAMG to be utilized in ocean monitoring systems, it must operate in diverse environments. In the ocean, the salinity concentration ranges from 3 wt.% to 4 wt.%, and in specific conditions such as the Dead Sea and glaciers, the NaCl concentration can vary between 3 wt.% and 30 wt.%. Furthermore, in such environments, temperature fluctuates within a range of 0–40 °C. Under these conditions, the proposed VAMG demonstrates consistent performance (Figures S18 and S19, Supporting Information). In addition, we conducted a time‐dependent stability test under the extreme environmental conditions. The VAMG was tested under high salinity (30 wt.%) and temperature conditions (0 °C) for 7 days. The device continued to operate with stable performance throughout the testing period (Figure S20, Supporting Information). We also verify that its performance remains stable even after 90 days of continuous use (Figure S21, Supporting Information).

### 2.4 Wave Monitoring Under Ocean Conditions and Frequency Domain Comparison with Conventional Self‐Powered Ocean Monitoring Sensors

**Figure 5:**
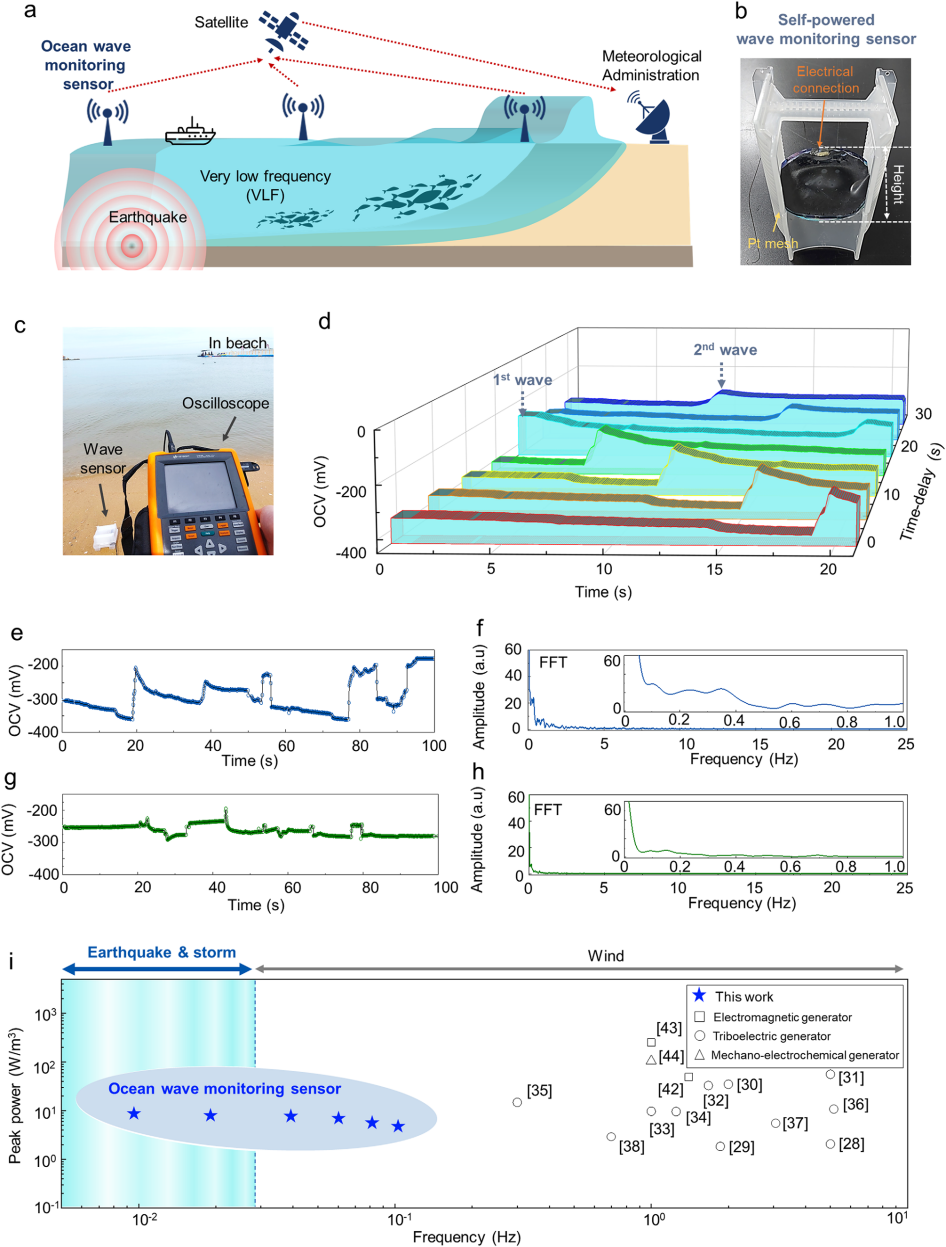
Self‐powered wave sensing performance in real ocean wave. a) Schematic diagram of an advanced self‐powered ocean wave monitoring sensor capable of measuring ultralow frequencies and autonomously generating electrical energy. b) Self‐powered wave monitoring sensor using VAMG, which autonomously generates electrical energy in response to waves and measures their wave component. c) Photograph of ocean wave measurement at the beach using the wave sensor. d) Reconstruction of wave movements over time using the recorded electrical signals. e) Wave data measured by VAMG over 100 s at the beach, along with the f) FFT analysis of this data in the frequency domain. g) Wave data measured over 100 s at a breakwater, h) along with the FFT analysis of this data in the frequency domain. i) Comparison graph of the performance of conventional mechanical energy harvesters, such as electromagnetic, triboelectric, and mechano‐electrochemical generators, with VAMG, based on frequency. Wave frequency information below 0.02 Hz is closely related to phenomena like earthquakes and tsunamis, and detecting such low‐frequency movements without signal distortion requires advanced sensing technology.

We developed a self‐powered monitoring system that can operate semi‐permanently by generating its own electrical energy to address the need for a self‐powered system (**Figure** **5**a; Movie S2, Supporting Information). This system comprises a circular VAMG (working electrode) integrated with a plastic frame and a Pt mesh as the counter electrode (Figure 5b; Figure S21, Supporting Information). The generated voltage was measured using an oscilloscope. To verify whether the system can capture actual wave data, we conducted measurements at Eurwangni Beach at 9 AM, with the sea temperature at 27°C.

The results indicated that the potential difference was generated according to the shape of the waves, which allowed us to reconstruct a 3D image of wave patterns over time (Figure 5c,d). In addition, we analyzed the shape of the waves according to location by measuring waves at the shallow beach (Figure 5e,f), including the deeper area near the breakwater (Figure 5g,h). The wave height was more pronounced at the beach, which led to a more significant OCV difference and an increase in FFT amplitude compared to the breakwater area (Figure S23, Supporting Information). The frequency domain analysis using FFT demonstrated that both signals shared a similar trend despite differences in amplitude. The VAMG is well‐suited as a wave monitoring sensor because it can capture low‐frequency motion. Although high‐frequency waves are typically generated by wind, ultralow‐frequency waves below 0.02 Hz are triggered by underwater earthquakes and typhoons,^[^ ^5^ ^]^ which implies that the ability to measure such information is crucial.

Compared to conventional mechanical energy harvesters for ocean monitoring systems, the proposed VAMG demonstrates distinct advantages in ultralow frequencies (Figure 5i; Table S1, Supporting Information).^[^ ^4^, ^26^, ^27^, ^28^, ^29^, ^30^, ^31^, ^32^, ^33^, ^34^, ^35^, ^36^, ^37^, ^38^, ^39^, ^40^, ^41^, ^42^, ^43^, ^44^ ^]^ Piezoelectric and triboelectric energy harvesters operate optimally at higher frequencies, generating up to 55.6 W m^−^^3^; however, their performance declines significantly at low frequencies. Although a few studies utilizing ball‐shell structures have produced 2.06 W m^−^^3^ at frequencies below 5 Hz, measuring movements in extremely low frequencies, particularly below 0.02 Hz, remains challenging due to fundamental limitations. In addition, ball‐shell structures, in which an internal ball moves with wave motion to generate electrical energy, are limited in capturing precise wave information like wave height and frequency. An alternative approach is developing hybrid ocean monitoring systems that combine triboelectric and electromagnetic generators. These systems, which employ swing‐tube structures, achieved a high performance of 18.98 W m^−^^3^ at a low frequency of 1.4 Hz. However, structures that generate electricity based on the oscillation of internal fluids remain limited in measuring low‐frequency motion accurately and obtaining detailed wave information.

In contrast, a mechano‐electrochemical energy harvester has demonstrated a high output of 104.5 W m^−^^3^ at frequencies below 1 Hz. This harvester comprises a coiled CNT yarn fabricated by extreme twisting insertion into the CNT yarn. When the yarn is stretched, the internal CNT bundles experience a squeezing effect, converting mechanical energy into electrical energy. However, its sensing capability in the extremely low‐frequency range below 0.02Hz remains unexplored in the literature. Moreover, inherent issues in 1D coiled fibers, such as self‐untwisting, snaring, and a high Young's modulus (over 700 MPa), limit their ability to measure movements of compressible fluids like ocean waves accurately.

The proposed VAMG addresses these limitations by utilizing fluctuations at the electrode‐electrolyte interface, presenting a novel mechano‐electrochemical energy harvester based on unique structural and material advancement. By exploiting capacitance alterations from immediate electrode‐electrolyte interface fluctuations, the proposed VAMG can continuously measure actual slow movements below 0.01 Hz, minimizing interference caused by signal degradation or distortion from packaging or high Young's modulus. Furthermore, the 2D planar structure mitigates structural instability issues, such as self‐untwisting and snarling, enabling it to be freestanding and suitable for diverse applications.

## 3 Conclusion

The proposed VAMG system exhibits excellent potential as an ultralow‐frequency energy harvester and self‐powered wave monitoring sensor. Utilizing the electrochemical interaction between the CNT surface and electrolyte, it effectively generates electrical energy from the mechanical motion of slow ocean waves. This enables the VAMG to function efficiently in diverse environmental conditions, including actual ocean wave conditions, without signal distortion. The obtained results demonstrate the VAMG's ability to capture and analyze complex waveforms with overlapping frequencies, which is crucial for understanding natural phenomena such as tsunamis or seismic sea waves.

Currently, vertically aligned carbon nanotube forests are expensive to purchase. However, the material we developed costs only 0.365 USD cm^−^^2^, and we believe the price can be further reduced with mass production. To improve the efficiency of the VAMG, further studies on enhancing the electrochemical capacitance through surface modification are required. Therefore, we plan to investigate the chemical and electrochemical surface functionalization of carbon nanotubes, as well as the replacement of silicone rubber substrates with materials bearing various functional groups. Additionally, we aim to explore the synergistic effects of combining these two strategies to enhance device performance. Also, we expect to expand this research beyond simply detecting ocean wave motions. Our future work will focus on developing sensors capable of monitoring environmental changes such as earthquakes and disturbances in rivers and lakes. Furthermore, we envision applying this technology to the biomedical field, particularly for detecting subtle fluidic movements to monitor physiological conditions in the human body.

## 4 Experimental Section

### Fabrication Process

A solution fabricated by mixing a silicone rubber compound and a curing agent in a 1:1 ratio was evenly applied (20 g) to the forest surface. Due to the silicone rubber's high viscosity, some parts of the solution infiltrated between the CNT and solidified, forming a VAMG with an average thickness of 2 mm.

### Artificial Wave System

A 1 × 7.5‐cm VAMG was connected to an electromagnetic motor. Using a control program, the water level was adjusted. The system allowed frequency variations in the 0.01–1 Hz range and measured the water level in increments of 0.2 cm at an electrolyte temperature of 30 °C. The solution concentration employed for the experiments to simulate ocean conditions was 0.6M NaCl. To generate artificial waves, a 250‐ml beaker with 50‐g weights was utilized to create low‐frequency waves in the range of 0.04–0.08 Hz, generating water levels of 0.4–1.2 cm. The distance from the wave center to the VAMG was 5 cm. To generate interfering waves with multiple sine waves, a 30 × 80 cm tank was filled with an electrolyte to a 20‐cm height. Artificial waves were generated using a fan 80 cm away to create wind and waves. These waves collided with the walls, overlapping multiple sine waves to form interfering waves. Waves with height and period of 1 cm and 0.1 Hz, respectively, were repeatedly applied. The difference between the maximum and minimum potentials was measured. Considering real seawater salinity ranging from 3.1 wt.% to 3.9 wt.%, the maximum salinity was set to match the Dead Sea's concentration of 30 wt.%. Experiments were conducted from 3 °C to 40 °C to simulate polar and tropical ocean conditions.

### Real Ocean Wave Measurement

Real waves were measured at Eurwangni Beach with a water temperature of 27 °C at 9 AM. Two locations were selected: one near the shore and the other in deeper waters near the breakwater. The working electrode utilized was a circular VAMG with a diameter of 10 cm, and the counter electrode was a Pt mesh. Measurements were taken using a portable oscilloscope.

### Characterization

The electrochemical performance was evaluated using a Gamry G750 potentiostat with a three‐electrode setup comprising a working electrode, a Pt mesh counter electrode, and an Ag/AgCl reference electrode. To measure electrical power and energy, the sensor was connected to an external resistor, and the voltage across the resistor was recorded using an oscilloscope (Tektronix, DPO4014B) while varying the resistance. Electrical power was calculated using the Equation (3),

(3) $$P=V^{2}/R$$

where P, V, and R denote power, voltage, and resistance, respectively. Morphological analysis was conducted using a field‐emission scanning electron microscope (FESEM, Hitachi S4700, Japan) operating at 15 kV. Electrical measurements were taken with a digital multimeter (Model 187, Fluke Corporation, USA). Mechanical testing was performed with a universal testing machine (INSTRON 5966, USA).

### Statistical Analysis

Experimental data were collected from at least five independent samples (n = 5) and are presented in the figures as mean ± standard deviation.

## Conflict of Interest

The authors declare no conflict of interest.

## Supporting information



Supporting Information

Supplemental Movie 1

Supplemental Movie 2

## Data Availability

The data that support the findings of this study are available from the corresponding author upon reasonable request.
