# Supplementary material for: Vertically Aligned Carbon Nanotube Mechano‐Electrochemical Generator for Ultralow‐Frequency Ocean Wave Monitoring
Source: Adv Sci (Weinh). 2025 May 5;12(27):2503578. doi: 10.1002/advs.202503578 (PMC12279232; doi:10.1002/advs.202503578)
Supplement: Supplementary file 1 — Supporting Information [file ADVS-12-2503578-s002.docx]

Vertically Aligned Carbon Nanotube Mechano-Electrochemical Generator for Ultralow-Frequency Ocean Wave Monitoring

*Hyeon Jun Sim, Dong Yeop Lee, Hocheol Gwac, Seungjin Lee, Joonhyeon Jeon, Seon Jeong Kim, Young-Kwan Kim, Chang-Seok Kim, Young-Jin Kim,* Sooncheol Kwon,* and Changsoon Choi **

H. J. Sim

Department of Biomedical Engineering, Konkuk University, Chungju, 27478, South Korea

D. Y. Lee, H. Gwac, S. J. Kim, C. Choi

Department of Electronic Engineering and Biomedical Engineering, Hanyang University, Seoul 04763, South Korea

S. Lee

Department of Advanced Battery Convergence Engineering, Dongguk University-Seoul, Seoul 04620, South Korea

J. Jeon

Department of Electronic and Electrical Engineering, Dongguk University-Seoul, Seoul 04620, South Korea

Y. Kim

Department of Chemistry, Dongguk University-Seoul, Seoul 04620, South Korea

C. Kim

Department of Cogno-Mechatronics Engineering Pusan National University Geumjeong-gu, Busan 46241, Republic of Korea

Y. Kim

Medical Device Development Center, Osong Medical Innovation Foundation, Cheongju, Chungbuk 28160, South Korea

S. Kwon

Department of Energy and Materials Engineering, Dongguk University-Seoul, Seoul 04620, South Korea

*E-mail : Y. Kim (email: kim.yj@kbiohealth.kr), S. Kwon (email: kwansc12@dongguk.edu), and C. Choi (email: pccs2004@hanyang.ac.kr)

**Supplementary Table S1.** Comparison of frequency and power for present self-powered wave monitoring sensor with previous reported sensors.

|  | | Frequency (Hz) | Power (W/m^3^) | |
| --- | --- | --- | --- | --- |
| This work | Vertically aligned  MWNT generator (VAMG) | **0.01** | **5.25** |  |
| Previous self-powered wave monitoring sensor | Ball-shell structured TENG [28] | **5** | **2.06** |  |
|  | Spring-assist TENG [29] | **1.86** | **1.84** |  |
|  | Sandwich like TENG [30] | **2** | **34.65** |  |
|  | Soft-contact spherical TENG [31] | **5** | **55.6** |  |
|  | Encapsulated TENG [32] | **1.67** | **32.6** |  |
|  | Open-book like TENG [33] | **1** | **9.675** |  |
|  | Butterfly-inspired TENG [34] | **1.25** | **9.559** |  |
|  | Pendulum-structured TENG [35] | **0.3** | **14.71** |  |
|  | Buoy float TENG [36] | **5** | **14.3** |  |
|  | Spherical 3D TENG [37] | **2** | **8.3** |  |
|  | Ocean wave TENG [38] | **0.7** | **4.65** |  |
|  | Mechanical saker PENG [39] | **0.8** | **4** |  |
|  | PZT fiber PENG [40] | **2** | **0.118** |  |
|  | Plucking-driven PENG [41] | **40** | **390** |  |
|  | Swing hybrid electromagnetic nanogenerator [42] | **1.4** | **18.98** |  |
|  | Tube-shaped electromagnetic nanogenerator [43] | **1** | **250** |  |
|  | MWNT coiled yarn shaped mechano-electrochemical nanogenerator [44] | **1** | **104.5** |  |


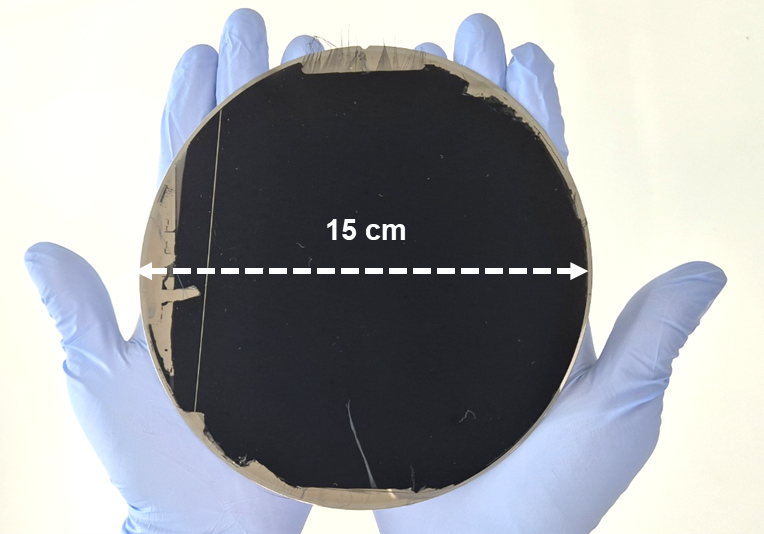


**Supplementary Figure S1.** optical image of 15cm-diameter vertically aligned MWNT forest.


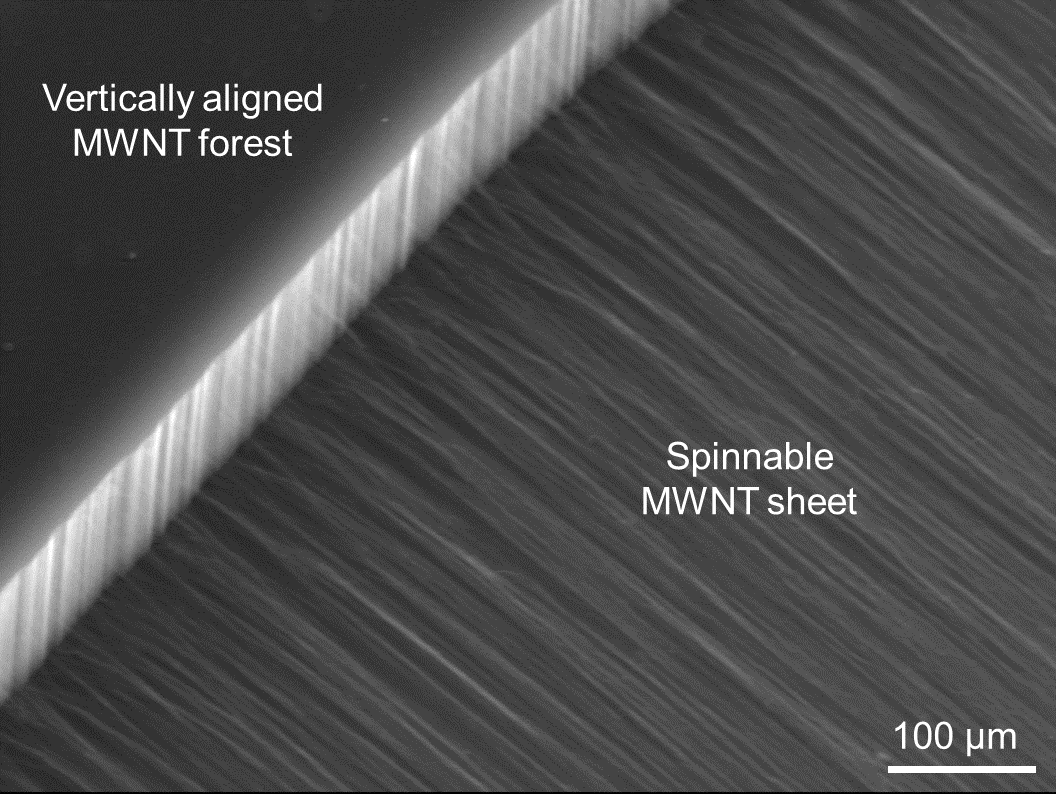


**Supplementary Figure S2.** SEM image of and spinnable MWNT sheet drawn from vertically aligned MWNT forest.


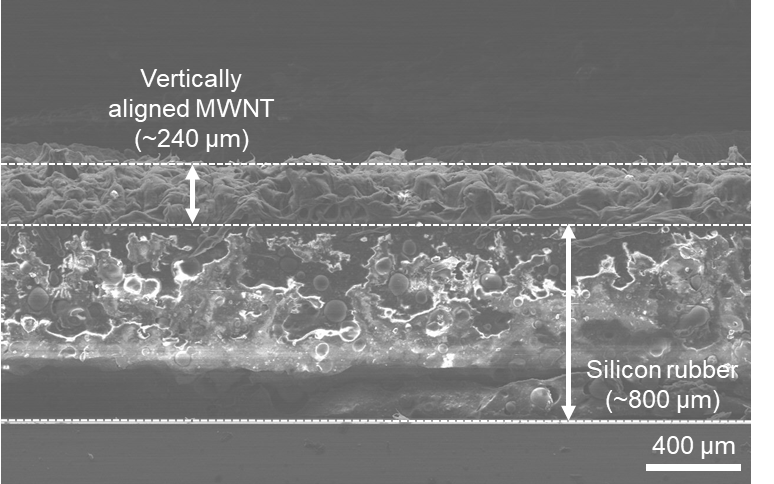


**Supplementary Figure S3.** SEM image of vertically aligned MWNT generator.


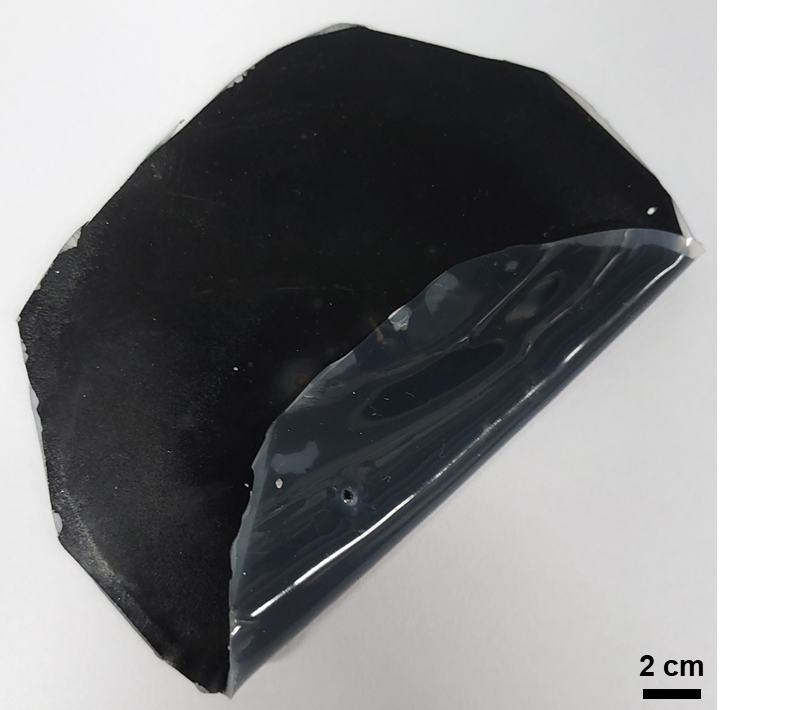


**Supplementary Figure S4.** optical image of 15cm-diameter vertically aligned MWNT generator.


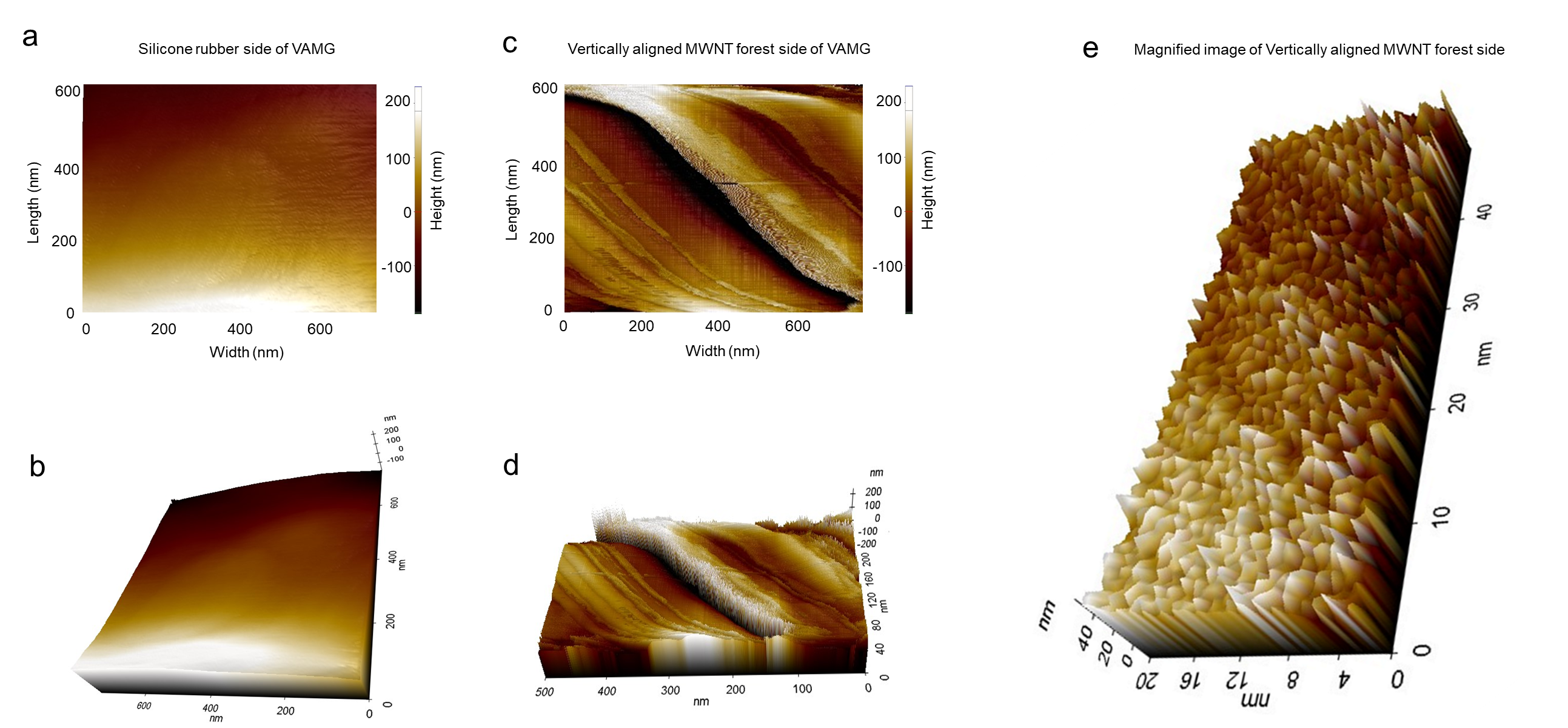


**Supplementary Figure S5.** The (a) AFM image and (b) magnified image of the MWNT forest surface of VAMG, the (c) AFM image and (d) magnified image of the silicone rubber surface.


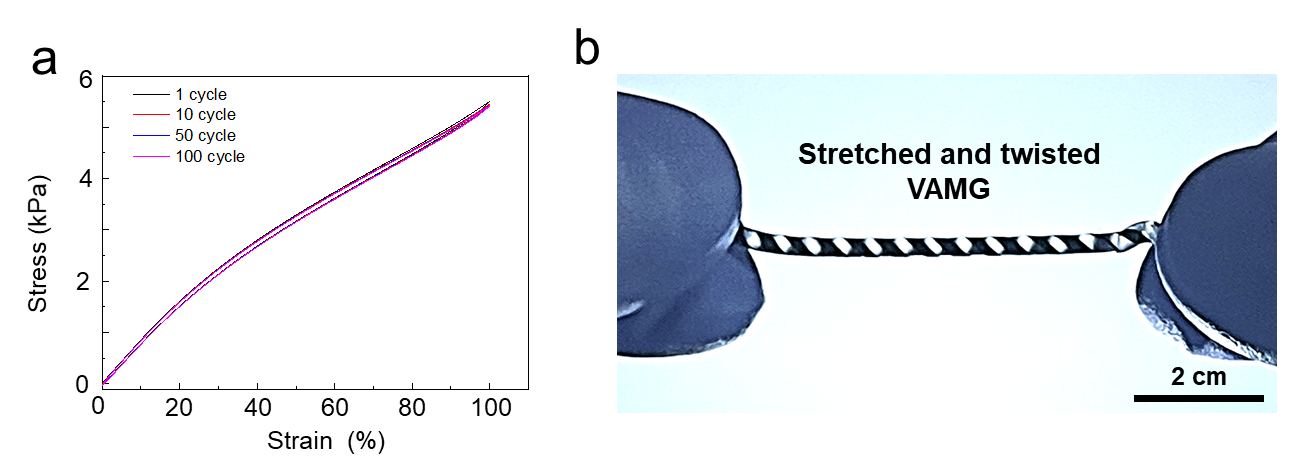


**Supplementary Figure S6.** (a) strain-stress curve of VAMGE. (b)The optical image of stretched and twisted VAMG


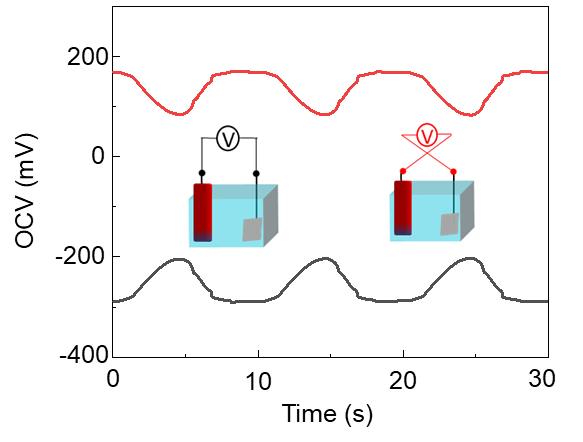


**Supplementary Figure S7.** Switching polarity test of VAMG (sign of generated OCV is reversed after the electrodes of measurement are turned over)


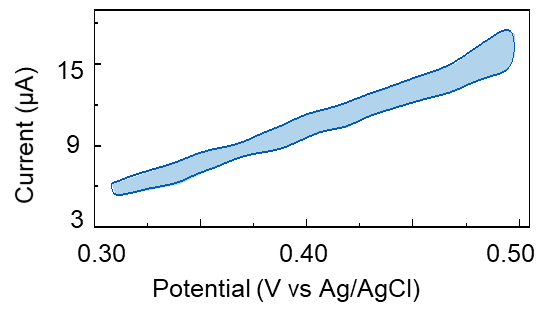


**Supplementary Figure S8.** Cyclic voltammograms of the VAMG electrode in 0.6 M NaCl with a immersed area of 0.1 cm² (scan rate: 100 mV/s).


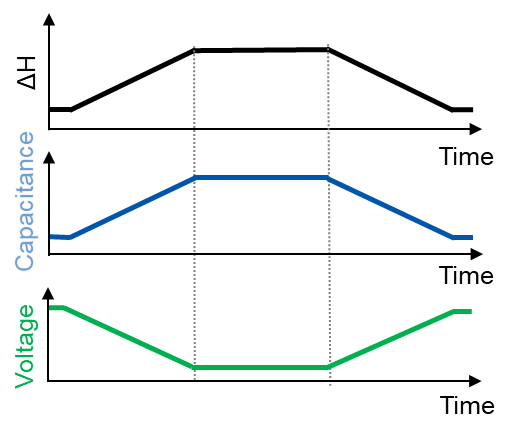


**Supplementary Figure S9.** The schematic diagram of wave amplitude(ΔH), capacitance and voltage of VAMG


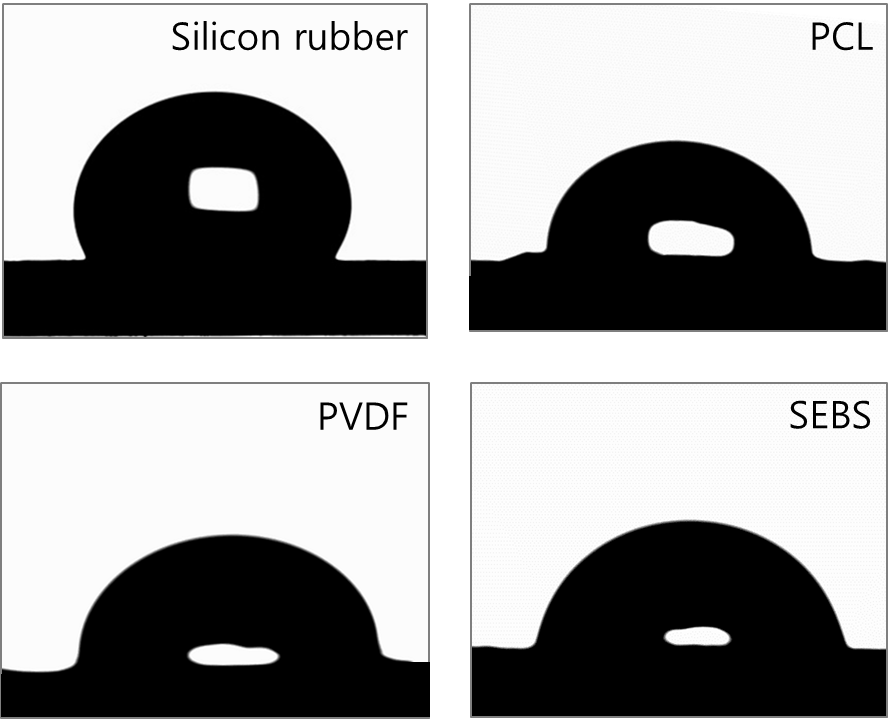


**Supplementary Figure S10.** The contact angle of MWNT forest/silicon rubber, MWNT forest/PCL, MWNT forest/PVDF and MWNT forest/SEBS.


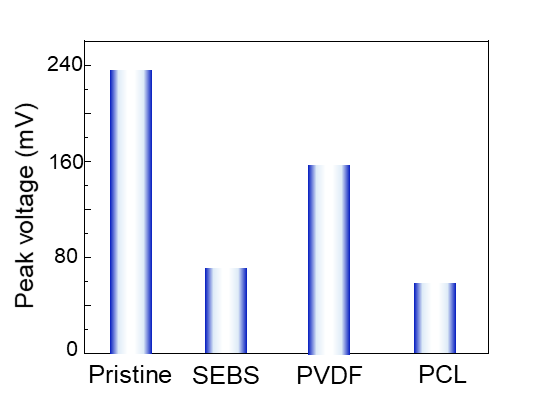


**Supplementary Figure S11.** The Peak voltage change of the VAMG, MWNT/SEBS, MWNT/PVDF and MWNT/PCL in 0.6 M NaCl with a immersed area of 1 cm²


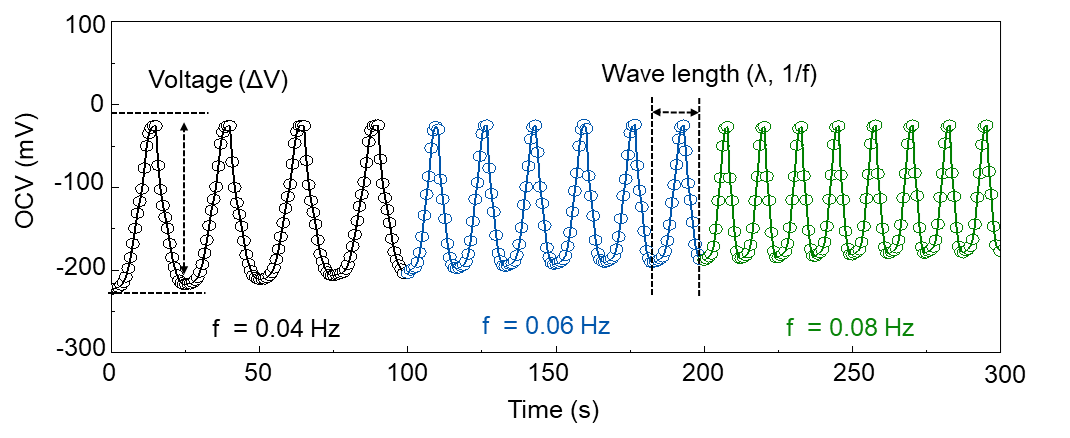


**Supplementary Figure S12.** OCV changes of VAMG with repeated immersion of a 1 cm² area in 0.6 M NaCl solution under mono-sinusoidal waves at low frequencies of 0.04, 0.06, and 0.08 Hz


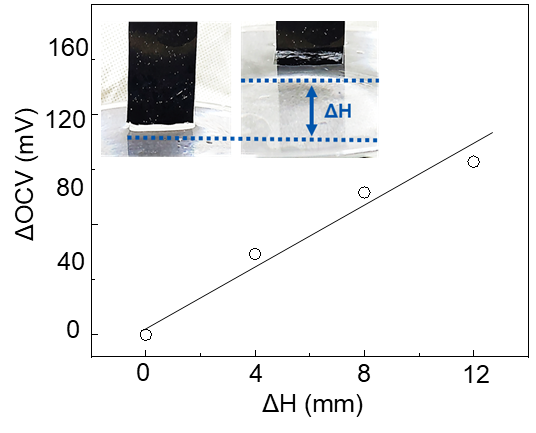


**Supplementary Figure S13.** OCV changes of VAMG when the immersion area increases up to 1.2 cm² in 0.6 M NaCl solution under a mono-sinusoidal wave at a low frequency of 0.1 Hz.


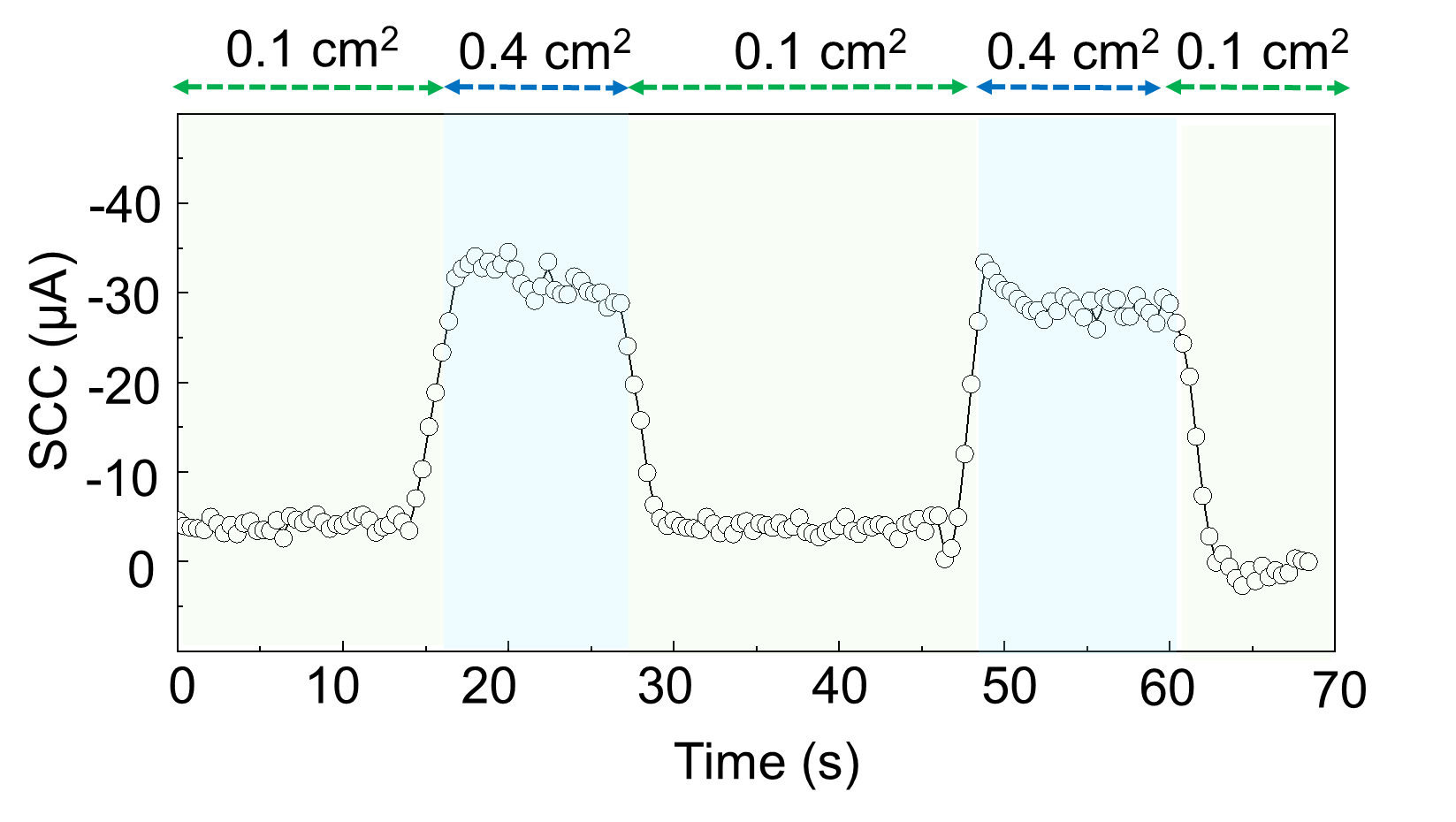


**Supplementary Figure S14.** SCC values generated by VAMG when subjected to a square wave stimulus with a 0.3 cm height.


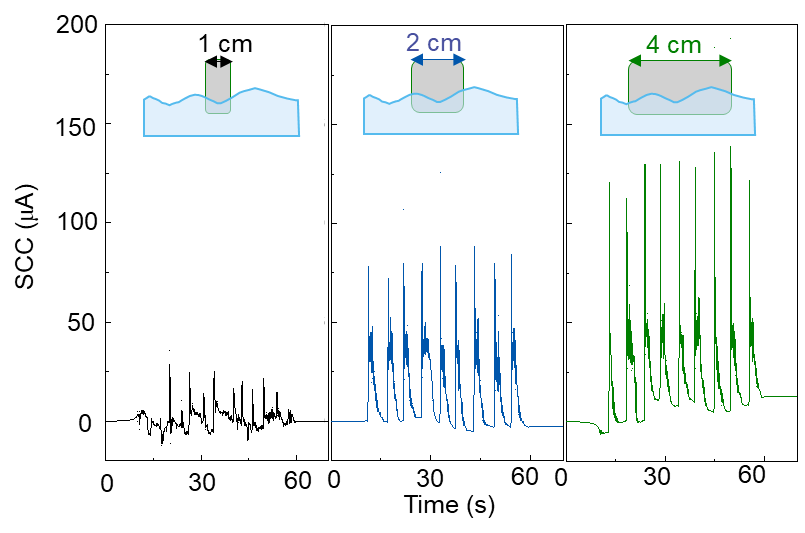


**Supplementary Figure S15.** SCC current generated by VAMG as its width increases from 1 cm to 4 cm under the same wave conditions.


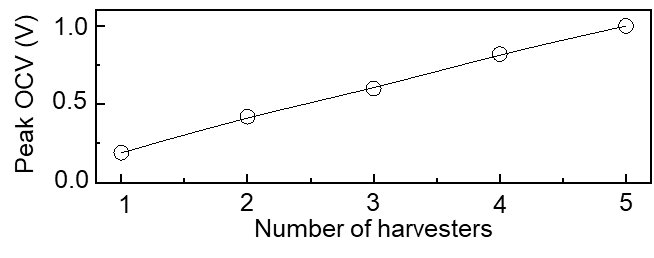


**Supplementary Figure S16.** Amplified OCV through the series connection of VAMG.


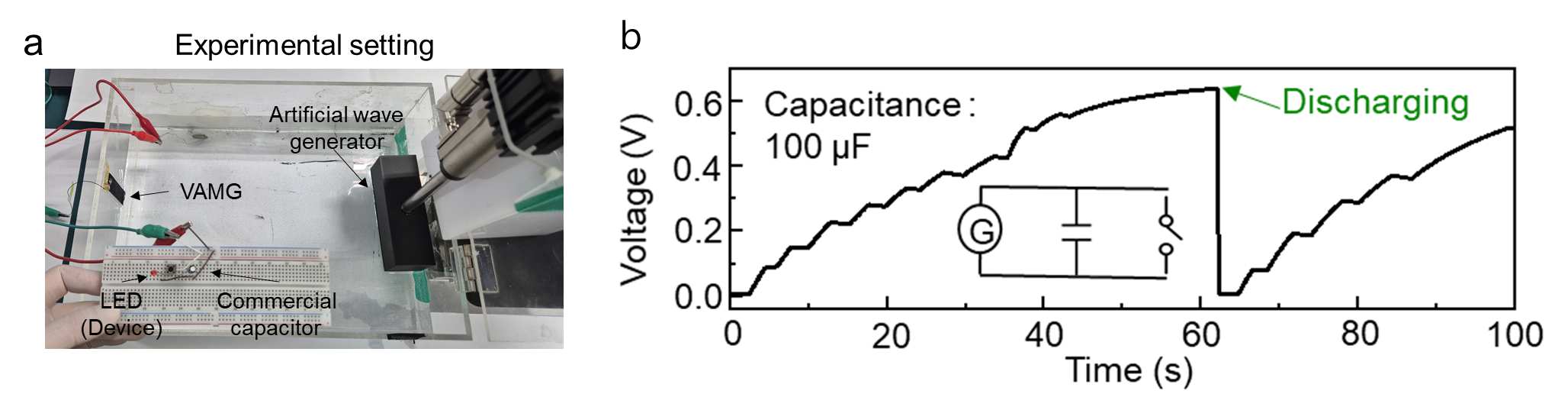


**Supplementary Figure S17.** (a) The optical image of experimental setting. (b) the generated electrical energy was charged into a 100µF commercial capacitor, and after discharging through a circuit, the capacitor was recharged


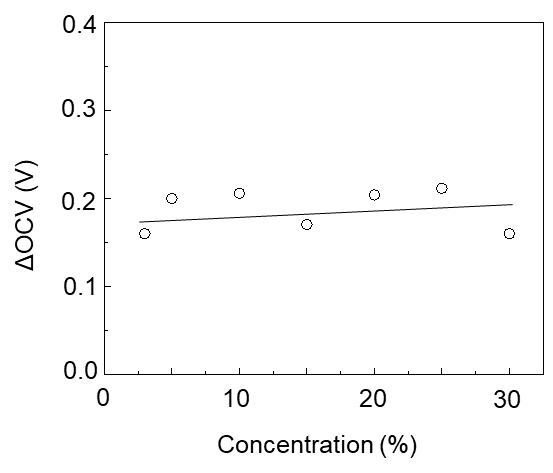


**Supplementary Figure S18.** VAMG output as the NaCl electrolyte concentration varies from 3 wt% to 30 wt%. The typical concentration of ocean ranges from 3 wt% to 4 wt%, while the Dead Sea has a high concentration of up to 30 wt%.


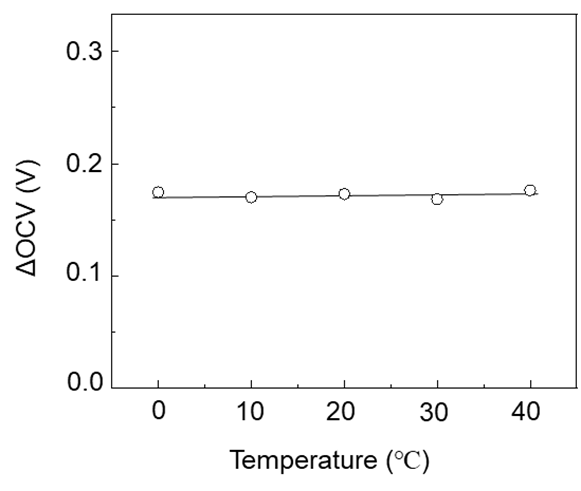


**Supplementary Figure S19.** OCV change of VAMG in 0.6 M NaCl as the temperature changes between 5°C and 40°C. The water temperatures in polar and tropical regions fall within this range.


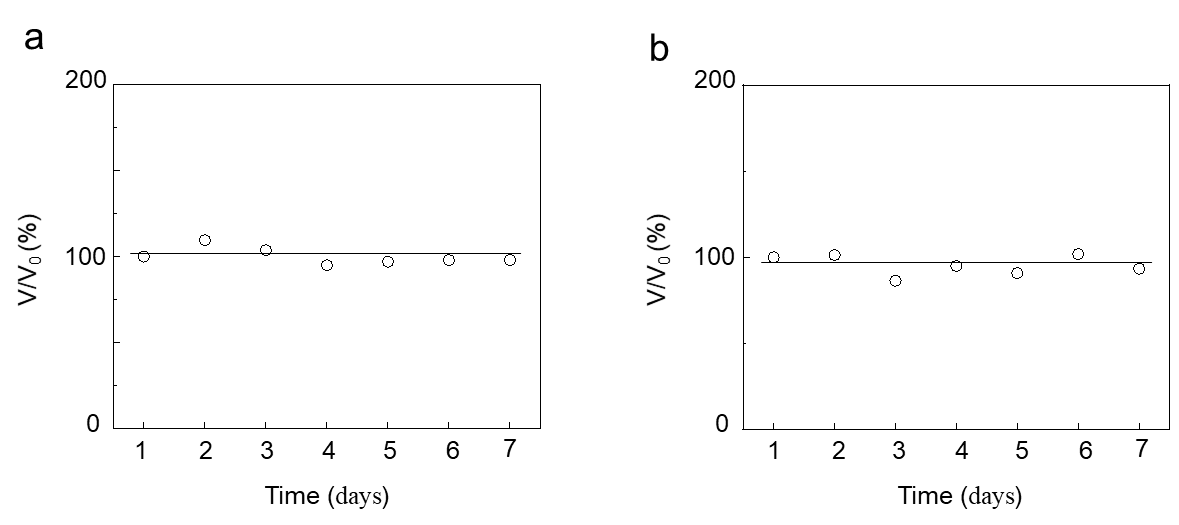


**Supplementary Figure S20.** OCV changes generated by VAMG over time in extreme condition of (a) at high salinity (30wt%) or extreme temperature condition (0°C).


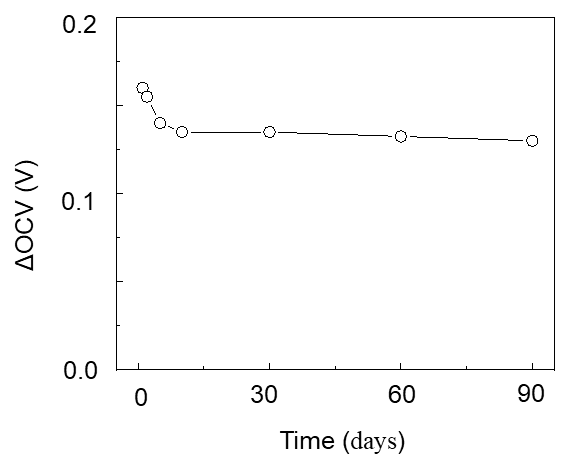


**Supplementary Figure S21.** OCV changes generated by VAMG over time, up to 90 days. After each time period, the average value was calculated from 100 cycles.


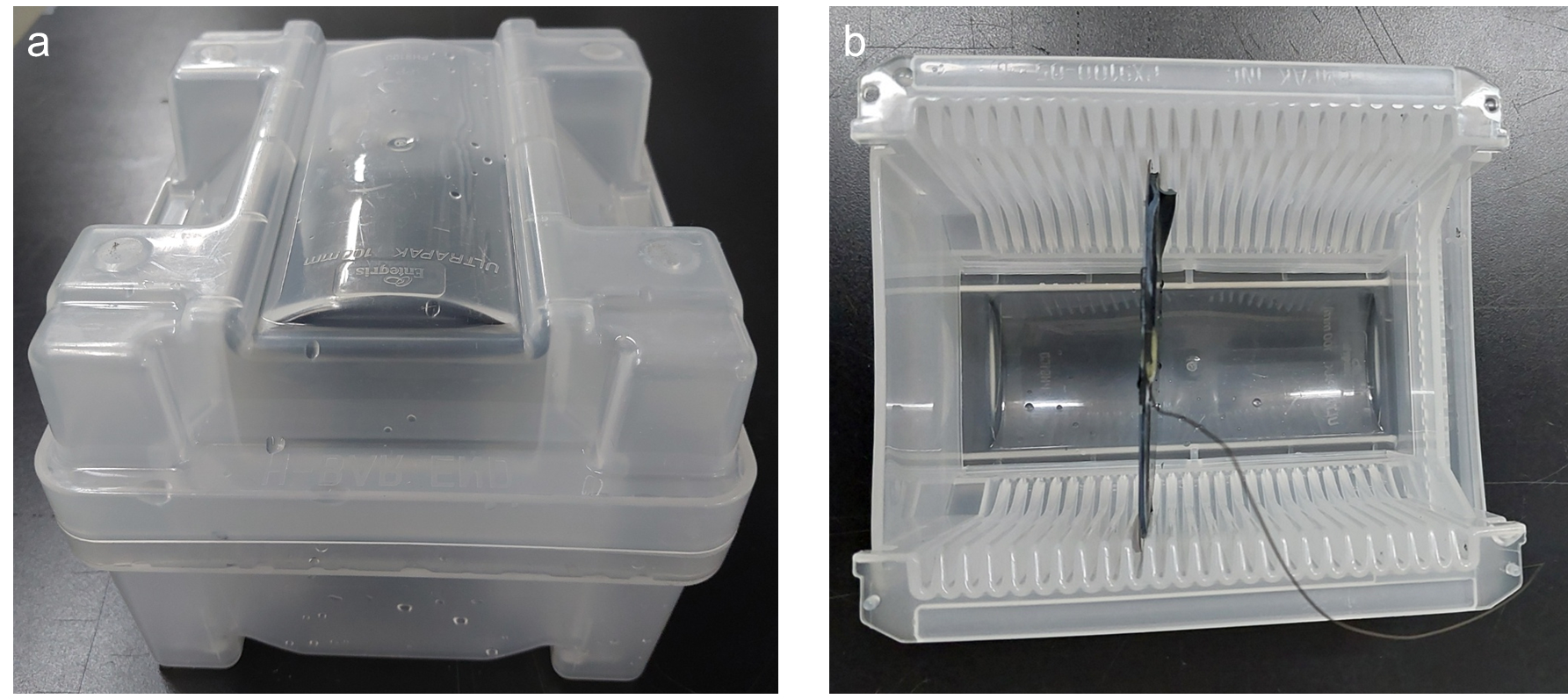


**Supplementary Figure S22.** Optical image of the packaging for an ocean monitoring sensor. To minimize mechanical disturbances and maximize the measurement of water flow, a channel-type device was developed, allowing seawater to pass through.


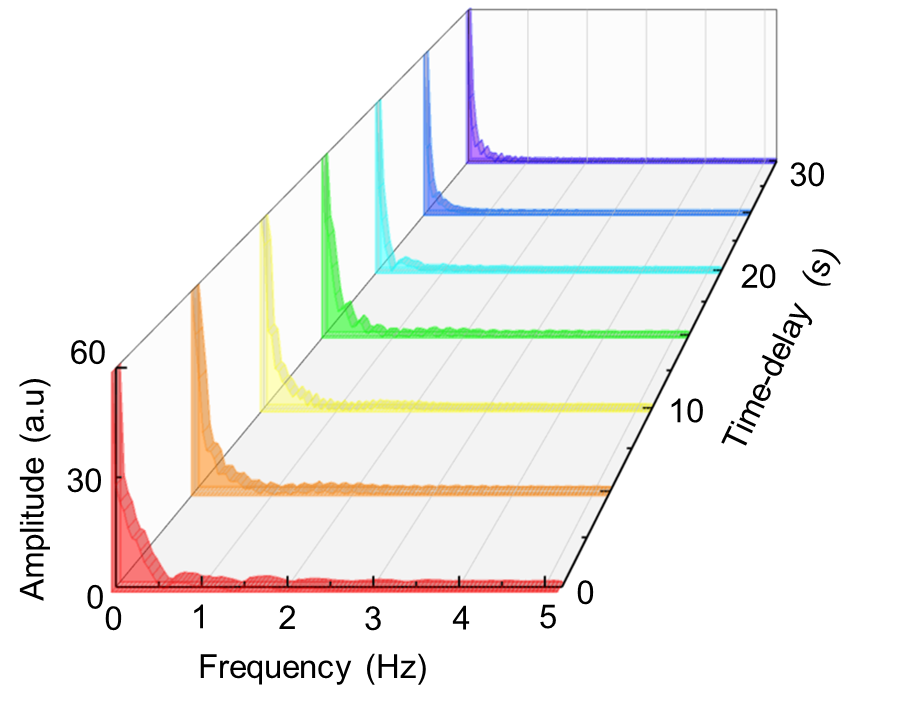


**Supplementary Figure S23.** The waveforms measured by the oscilloscope were continuously subjected to FFT every 20 seconds to compare and analyze the frequency components of the waves over time.
